# Counting within the Subitizing Range: The Effect of Number of Distractors on the Perception of Subset Items

**DOI:** 10.1371/journal.pone.0074152

**Published:** 2013-09-16

**Authors:** Liat Goldfarb, Sharon Levy

**Affiliations:** E.J. Safra Brain Research Center for the Study of Learning Disabilities, Department of Learning Disabilities, University of Haifa, Haifa, Israel; University of Melbourne, Australia

## Abstract

When exploring the mechanisms involved in perceiving numbers we must distinguish between two types of numbers: subset numbers (e.g., perceiving "2" when two plates and one cup are displayed on a table) and the total number of items (e.g., perceiving "3" objects in the previous example). Combining feature perception theories with number perception theories, the current paper explores the mechanisms involved in the perception of small numbers in feature-defined subsets. The paper introduces several theories for how subset items can be represented and examines an important prediction of those theories: Will the number of distractors affect the perception of small subset items? In two experiments, we found that the response time (RT) for counting small target items that differ from their distractors by a single feature was faster when there were few distractors compared to many distractors. This was found for different types of distractors: distractors within and outside the subitizing range. Only when distractors were organized in a specific pattern, allowing distractor grouping, the increase in the number of distractors did not affect target counting. The current study suggests that even when performing simple counting of subset targets, the enumeration process can begin only once the locations of the targets have been identified and the targets' shape is bound to these locations. This pre-counting procedure depends on the number of individual locations occupied by the distractors. These findings are further discussed within the context of the object file theory.

## Introduction

The ability to perceive quantities exists not only among human adults but also in some form among infants and animals [1]–[2]. It has been shown that even very young infants can discriminate between quantities. The perception of quantities develops gradually throughout infancy, long before children learn to count verbally or to identify the arithmetic symbols [3]–[4]. Kaufman, Lord, Reese and Volkmann [5] named the perception of a small number of items "subitizing" (from the Latin word "subitus" meaning "sudden") to reflect the notion of one's sudden perception of small numbers. This type of perception relates to the processing of one to three or four items. Many studies show that typically developed children and adults perceive quantities within the subitizing range differently than quantities beyond that range. For example, numbers within the subitizing range are perceived more rapidly and accurately than quantities larger than four [6]. In addition, in comparison to the subitizing range, steeper response time slopes are observed for items beyond the subitizing range. That is, beyond four items, the response time significantly increases linearly with each item added [7]–[9]. This is thought to suggest that unlike numbers within the subitizing range that are perceived rapidly, numbers larger than four require serial processing.

It has been proposed that a parallel pre-attentive process is required for perception of numbers within the subitizing range, whereas numbers beyond the subitizing range are connected to a serial attentive process. Piazza, Giacomini, Le Bihan and Dehaene [10] in an fMRI study found a sudden increase in the activity of attention related areas in the posterior parietal and frontal cortices, only for displays that contained four or more items. These results indicate that processing numbers beyond the subitizing range requires greater attention resources than those needed for perception of numbers in the subitizing range. On the other hand, other studies suggest that subitizing depends to some extent on attentional resources [11]–[15].

In an article recently submitted for publication we [16] discussed the role of attention resources in small number processing. We explored the perception of a total number of items in a display in comparison to the perception of the number of subset items within that total. We suggested that perceiving the number of items in a subset (e.g., perceiving "2" when two plates and one cup are displayed on a table) is qualitatively different than perceiving the total number of items (e.g., perceiving "3" in the previous example). This kind of number of subset perception might occur via an attentional path and it might be an effortful process. At an early perception stage, features in our surroundings such as size, color, and shape are perceived in their special feature maps rapidly, simultaneously, and without utilizing attentional resources [17]–[19]. Detecting numbers in a subset adds a perceptual problem to the enumeration procedure - the problem of detecting identical elements. In order to individuate identical items, a representation must be created in which each individuated location is bound to the specific identity. According to this notion, a subset enumeration requires the creation of a mental representation in which the identity of the items, such as their shape, must be perceived and distinguished from one another in order to enable enumeration. Unlike perception of the total number of items, which only requires knowledge about the location of items (and not their other features), counting a subset first requires the identification of each item presented in relation to the relevant feature that is being counted (e.g., a certain shape). In other words, perception of the number of subset items occurs only after the creation of a mental representation in which individual locations and the relevant identity (e.g., shape) of the items in these locations are already known. In line with this suggestion, Goldfarb and Treisman [16] found that binding errors that characterize the binding of different features of items (such as color and shape) are not present when the binding is between the shape of the items within a subset and their number. These results indicate that while the perception of features such as color and shape occurs simultaneously, the perception of the number of a subset is not concurrent with the perception of the shape of the subset items, but rather follows it via an attentional binding procedure.

Exploring the perception of subset numbers is of importance since it has been found to be related to other mathematical abilities in school learning. For example, Halberda, Mazzocco and Feigenson [20] found a correlation between the acuity of the approximate number system (ANS), measured by performance on a task that requires the perception of subset, and symbolic math performance. Their results show that perception of subset in the ninth grade was correlated with symbolic math performance of individual students from as early as kindergarten. In addition to its relevance for math abilities, the perception of subset numbers is necessary in everyday life, when objects usually appear as part of an overall group of items. In everyday life we rarely have need to simply enumerate the number of "things" (items whose identity is irrelevant). However, we often need to enumerate pre-defined subset items from among a total number of items (e.g., enumerate the number of cups on a table laid with both cups and plates).

### The current study

In our previous study, we [16] explored the perception of subset number by addressing the phenomenon of binding errors. In the current paper we explore a new question related to the perception of subset numbers by addressing a different perceptual phenomenon: the interference of the number of distractors. As noted previously, in Goldfarb and Treisman [16] we suggested that perception of the number of subset items occurs only after the creation of a mental representation in which the individual locations and the shape of the items are already bound. An important question arising from this notion is what exactly is the form of this mental representation and, specifically, how do the main characteristics of this mental representation affect the counting of a select subset (i.e., when one must count a certain subset while ignoring other items).

**Figure 1:**
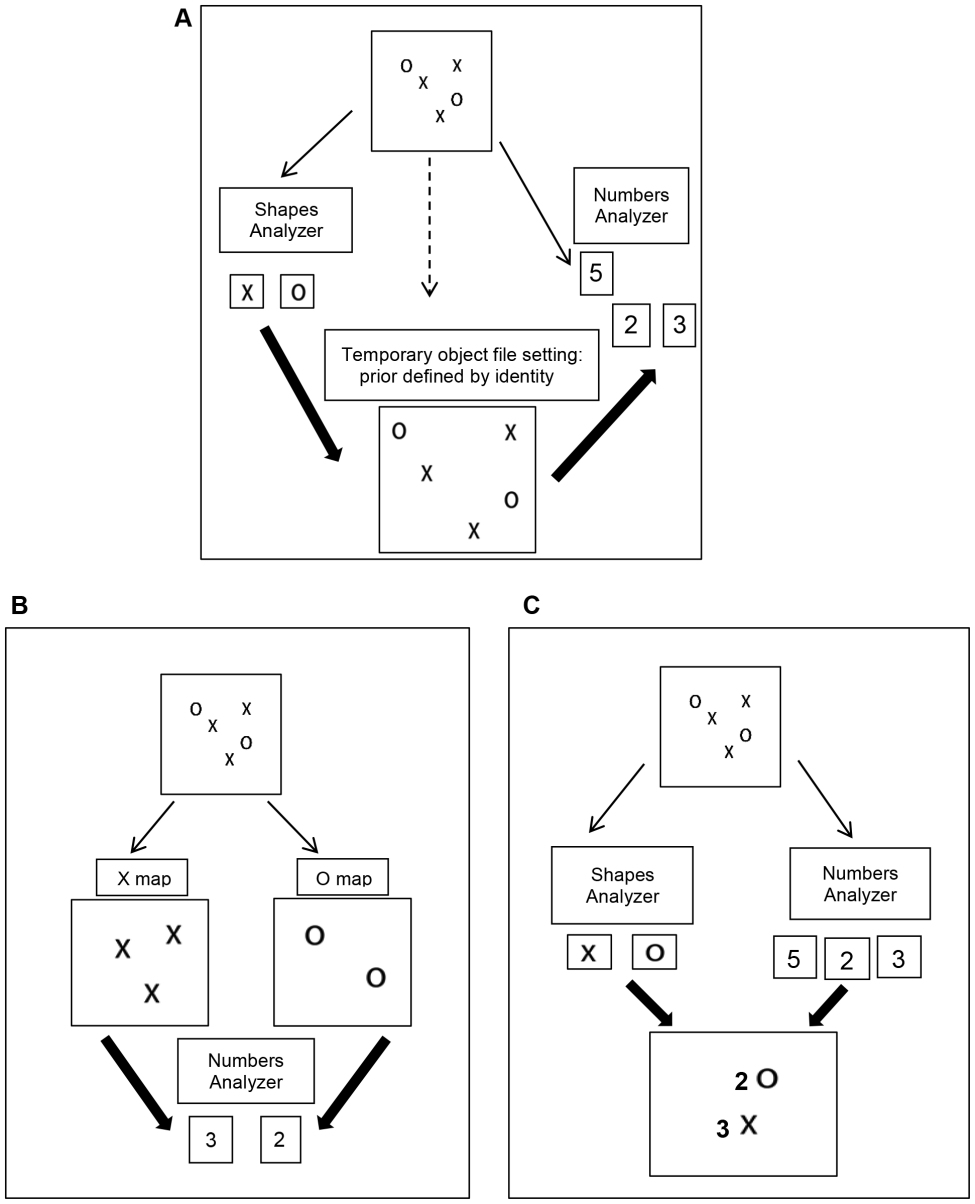
How can subset items be counted? (A). Option 1: Counting subsets based on the object file representations where counting occurs only after the creation of a mental representation in which all possible locations are checked and the shape of the items is bound to the relevant location. (B). Option 2: Several identical items are represented in one single map. The number of a subset could be directly pulled out of that map without checking all possible locations. (C) Option 3: The arithmetic system perceives the number of a subset as a feature, similar to the way other systems perceive other features such as color or size.

One option of mental representation in which the system can count the number of subsets and suggested in our two recent studies [16], [21] is illustrated in Figure 1a. According to this option each instance (such as the features X or O) is stored in the recognition system only once. When identical objects appear simultaneously (e.g., many Xs among Os) and one has to count target items (e.g., Xs) the system must check the shape of each location in the surroundings and then match and bind the shape of the target items to the relevant location. After this stage is performed the arithmetic system can enumerate the number of target subsets. This option is similar to that reflected in the object file theory [22] and it also has a clear prediction regarding distractor interference when counting subsets. The theory predicts that the more distractors there are the more demanding the perception of the number of targets.

Another option is that the number of a subset could be directly pulled out of the relevant feature map, such as the shape map (see Figure 1b). According to the Boolean map theory [23], at an early perception stage, each instance of a feature (e.g., the instance red and the instance green) is represented in a separate map (i.e., a red map and a green map) and these maps cannot be accessed simultaneously. On the other hand, multiple locations of identical items (i.e., many reds) can be represented in a single map and be accessed simultaneously. One possible theoretical option that can arise from this kind of representation is that the number of a certain instance can be represented independently from the existence of other locations filled with other irrelevant distractors. Meaning that in a case where targets are identical instances that differ from the distractors only in one feature (i.e., reds among greens or Xs among Os) the number of different distractors will not interfere with the perception of the targets' number. Similarly, according to the notion of the activation map [24], when feature targets are searched for within a field of distractors, the relevant locations can be easily activated. Hence theoretically, the arithmetic system can directly enumerate these marked locations and the number of different distractors will not interfere with perception of the targets' number.

A third option (see Figure 1c) can be that at an early stage of perception, each subset number is perceived quickly and independently from among other subset numbers in a display. We noted that at an early perception stage, features in our surroundings such as size, color, and shape are perceived in their special feature maps rapidly, simultaneously, and without utilizing attentional resources [17]–[19]. For example when we see a red cup, the color area quickly processes the red color and is indifferent to the shape, while the specialized shape area quickly processes the cup shape and is indifferent to the color. The binding between the color and the cup occurs only at a later perception stage. The same principle may apply to the processing of subset numbers. When we see 2 plates and 3 cups in a display we might first represent the arithmetical features “2” and “3”. In order to perceive that there are 2 plates and 3 cups in a later perception stage we attach the correct number to the shapes of the cups and the shapes of the plates. In this case, the arithmetic system might be the unit in which the arithmetical features (e.g., “2” and ”3”) are represented before any binding to other features takes place.

In Goldfarb and Treisman [16] we rejected this option in the context of binding error. We demonstrated that the number information (“3” or “2”) is not subject to the same perceptual illusions as other features, for example, forming illusory conjunctions similar to color, shape, and motion. However if this option was correct and numbers of subsets would independently "pop out" of a display, then this theory also has a clear prediction regarding another perceptual effect: distractor interference. According to this theory, similar to the second option that is based on Boolean maps, an increased number of distractors will not gradually increase reaction time (RT) for the enumeration of targets.

In conclusion, in this study we intend to expand the finding of Goldfarb and Treisman [16] in relation to the process involved in counting subset items (as described in Figure 1a) to another perceptual effect: distractor interference. Although in everyday life we often need to enumerate pre-defined subset items, in a surrounding that contains both targets and non-target items (e.g., enumerate the number of cups on a table laid with both cups and plates), this is the first study that is designed to directly address the role of the number of distractors in a feature subset counting.

## Experiment 1

**Figure 2:**
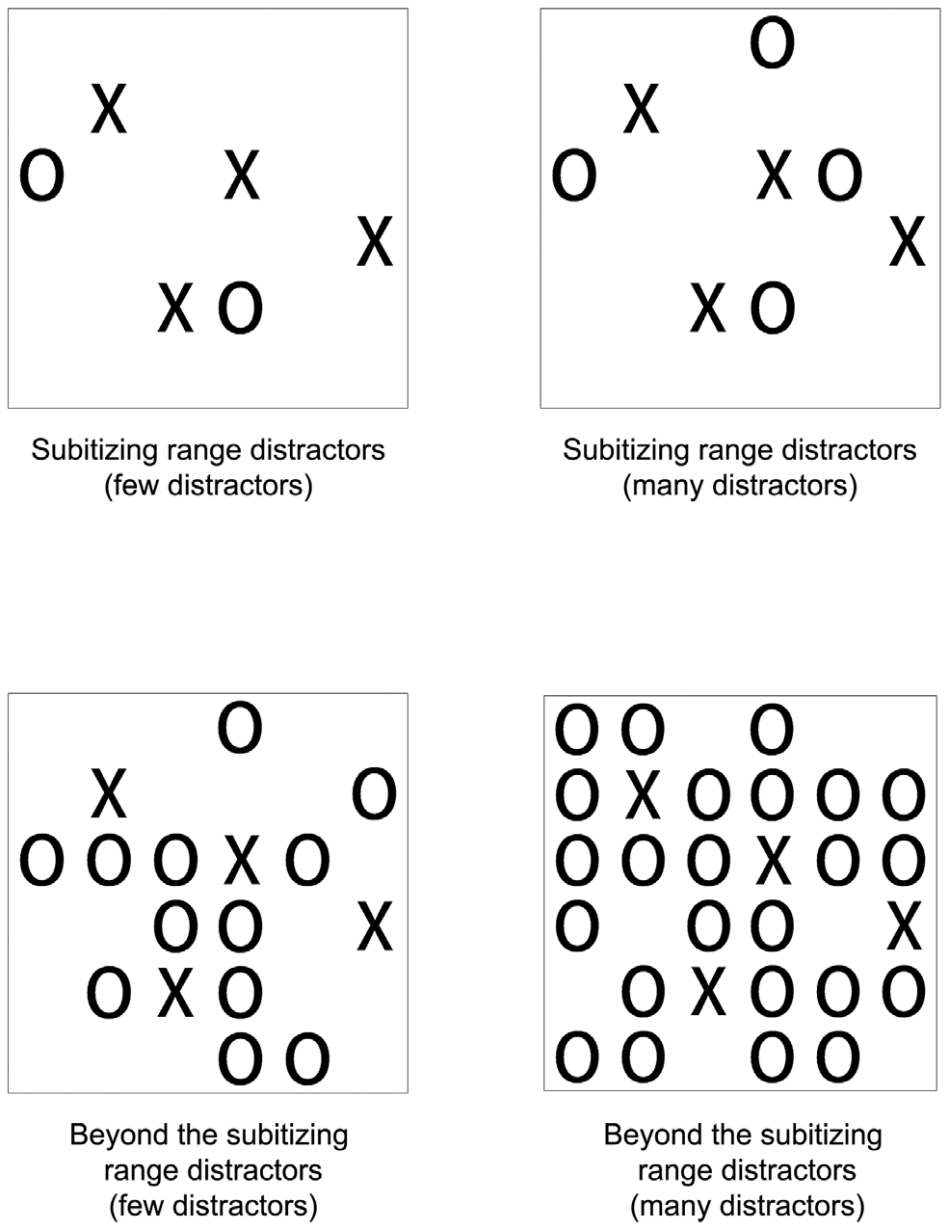
Examples of the displays in the various conditions in Experiment 1.

In Experiment 1 participants were asked to count the number of targets within the subitizing range while ignoring distractors. The distractors were either within the subitizing range or outside the subitizing range and they were either few or many (see Figure 2). If counting a subset depends on the prior binding between each possible location and its shape (such as the first option described in Figure 1a) then it is assumed that the RT for counting subset target items within the subitizing range will be faster in a display with few distractors than in one with many distractors. This pattern should be observed regardless of the nature of the number of distractors (i.e., whether the distractors are within the subitizing range or outside the subitizing range). On the other hand the number of distractors should not interfere with the perception of the targets' number if the number of a certain target can be directly pulled out of the scenery, or of a relevant feature map such as a shape map. This will also be the case if a representation in which the activation of all the locations of the feature target object pops out (such as the second and third option described in Figures 1b & 1c). In these cases the effect might also be determined by the nature of the distractors (i.e., distractors within the subitizing range that allow quick perceptions and are similar to the targets' number can be quickly pulled out of the display and might lead to a different effect than distractors that do not do so).

### Method

#### Participants

Sixteen adults between the ages of 18–26 (M = 20.75, SD = 2.15) participated in this experiment. All participants stated that they had no learning disabilities or attention deficits. The participants received credit points for their participation, as part of their obligations as first year students at Haifa University, or were paid 15 new Israeli shekels. Written informed consent was obtained from all participants. The Ethical Committee of Haifa University approved all the procedures in this study.

#### Measures

Stimuli were composed of black Xs (targets) and Os (distractors) placed on an invisible 6×6 object grid sized 8.5×5 cm. The size of each X and O was approximately 1×0.7 cm. Each stimulus contained either 3 or 4 Xs (targets). The experiment had two types of distractors: subitizing range distractors and beyond the subitizing range distractors. In the subitizing range distractors condition there were either 2 (few) or 4 (many) Os (distractors) (see Figure 2). For each combination of targets and distractors (3 Xs and 2 Os, 3 Xs and 4 Os, 4 Xs and 2 Os, 4 Xs and 4 Os) 8 different versions were created in which each distractor and target could appear in different locations on the grid. In total there were 32 different stimuli in this category. In the beyond the subitizing range distractors condition there were either 12 (few) or 24 (many) Os (distractors) (see figure 2). For each combination of targets and distractors (3 Xs and 12 Os, 3 Xs and 24 Os, 4 Xs and 12 Os, 4 Xs and 24 Os) 8 different versions were created in which each distractor and target could appear in different locations on the grid. In total there were 32 different stimuli in this category. For both subitizing range and beyond the subitizing range conditions for each of the 8 versions the targets' location on the invisible grid remained the same for all types of distractors (2/4/12/24 distractors).

### Procedure

The experiment was programmed on E-Prime 2.0. An HP Compaq computer with an Intel core i7–2600 central processor was used to present stimuli and to collect the data. Stimuli were presented on a 22 inch Samsung monitor, while participants sat at a distance of about 60 cm from the screen. A keyboard on which participants conveyed their answer was placed on a table next to the screen. Each participant was tested individually and the experiment took about 10 minutes in total.

The experiment began with a practice block that contained 16 stimuli presented randomly. Then an experimental block began. This block contained 64 stimuli, each presented three times at random (a total of 192 stimuli). Each trial began with a fixation that appeared for 512 ms, followed by the grid with targets and distractors that remained on the screen until the participant responded. All stimuli appeared in the center of the screen on a white background.

Participants were asked to report the amount of targets (Xs) in each stimulus as quickly and accurately as possible. They were asked to use their left index finger to press the number "3" and their right index finger to press the number "4", keeping both fingers on the keyboard at all times. Response time and accuracy were measured by the computer.

### Results and Discussion

Trials in which participants did not answer correctly (2.6%) and trials in which the RT was faster than 250 ms or slower than 2500 ms were not included in the analysis (0.7%).

For the remaining trials, the mean RT for each participant in each relevant condition was calculated. A two-way analysis of variance was applied to the mean RT with type of distractors (subitizing range/beyond the subitizing range) and number of distractors (few/many) as within participant factors.

**Figure 3:**
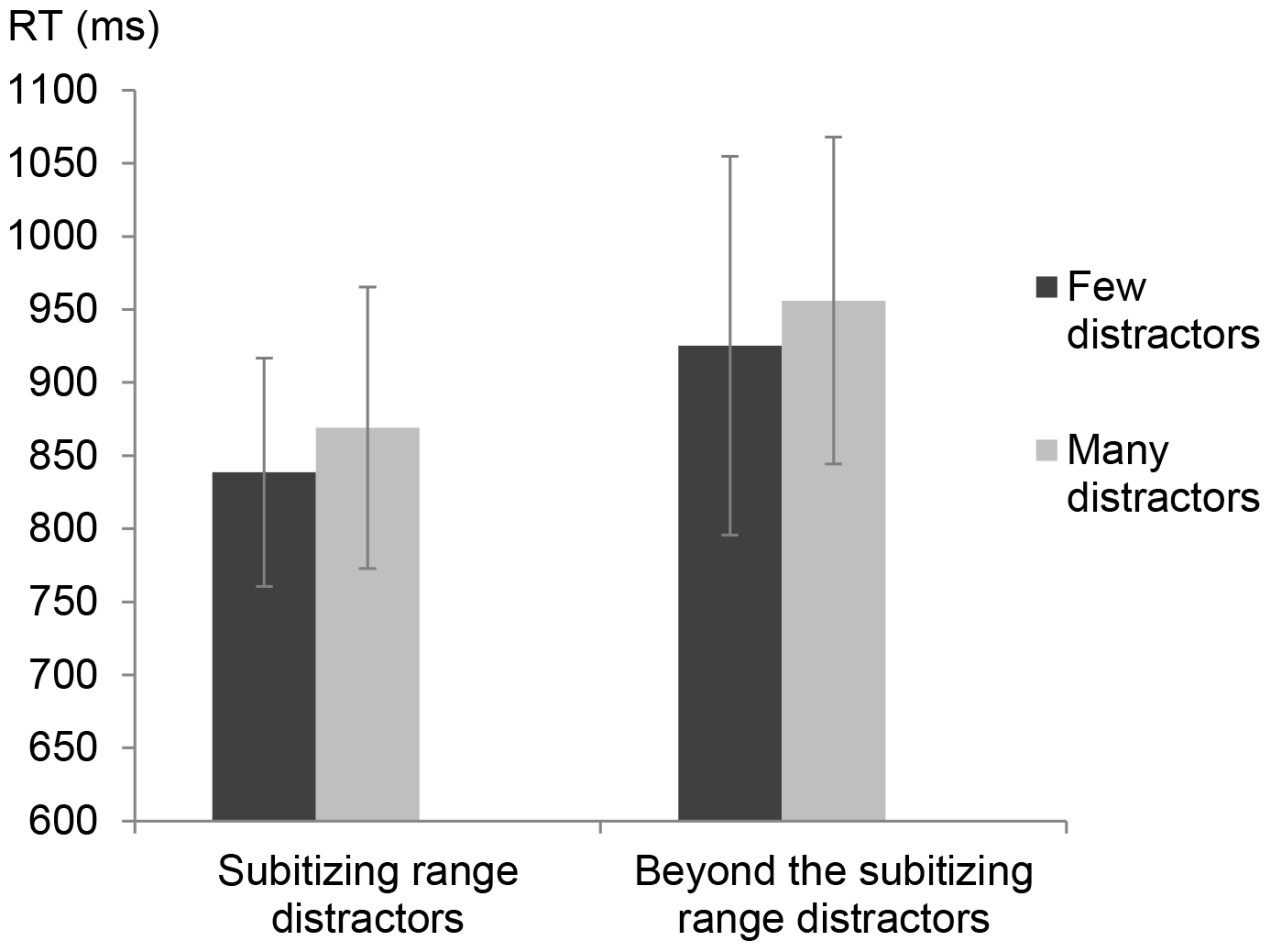
Mean RT and SD for counting subset items in the various conditions in Experiment 1.

The results revealed a significant effect for number of distractors (few/many), *F*(1,15) = 12.09, *MS_e_* = 1240, *p<*.01 and for type of distractors (subitizing range/beyond the subitizing range), *F*(1,15) = 56.93, *MS_e_* = 2118, *p<*.001. There was no significant interaction between the factors, (*F*<1). Further analysis revealed that when the distractors were within the subitizing range, RT for counting subset target items was significantly faster for few distractors (M = 838.68, SD = 78.15) than for many distractors (M = 869.08, SD = 96.23) [*t*(15) =  −2.17, *p<*.05]. These results were also found when the distractors were beyond the subitizing range, where the RT for few distractors (M = 925.28, SD = 129.49) was significantly faster than for many distractors (M = 956.12, SD = 111.84) [*t*(15) = −2.47, *p<*.05] (see Figure 3). Overall, the results indicate that RT for counting subset target items within the subitizing range became slower as the amount of distractors increased. This effect is not modulated by the nature of the distractors (i.e., whether or not the distractors were also within the subitizing range).

## Experiment 2

We noted that in everyday life objects usually appear in an environment together with other kinds of objects. Hence we often need to enumerate pre-defined subset items out of a total number of items. Experiment 1 showed that counting subset items depends on the number of distractors, meaning that the subset number is not simply pulled out independently of other distractors. However, under some perceptual conditions, subset items might be enumerated without checking the location of each distractor. For example when one has to count the number of apples on a tree that contains both apples and leaves, the setup of the leaves can affect the counting. The leaves on the tree could be organized in a way that creates a grouped single object with the tag "the crown of the tree". Hence in this setup, adding more leaves in a way that will be grouped with the existing tree crown will not add more distractions to the apple counting. In other words, the target search will not be conducted against more objects that interfere with the search. Hence in order to conclude if the number of distractors interferes with subset counting, we first need to identify what counts as an object that interferes with the counting. An object can be formed in many ways, of which grouping may be one [25]–[26].

Grouping is a common phenomenon in which elements are grouped together intentionally or automatically to form a single object (for a review see [27]). Several studies conducted with brain damaged patients suggest that grouping may prevent the item individuating process [28]–[29]. We have also recently suggested that in normal populations, the grouping of elements may form a single object even before the specific elements that construct it have been individuated [30]. In addition, the tag given to an object file based on grouped elements (i.e., the tag: crown of the tree) can replace the specific object tags that are given to the individuated items (i.e., each single leaf tag) [31]. Similarly, Treisman [32] found that in a search paradigm when the distractor items can be grouped, participants do not serially check the location of each distractor individually but rather refer to them as a group of distractors.

Hence, the described effect of RT increase for subset counting as a result of the increase in the number of distractors might depend on the distractors' organization. This effect might be reduced or even eliminated in an organized display of distractors that enables the additional distractors to be grouped together with the other distractors.

The following experiment is meant to test this assumption as well as to replicate the results of Experiment 1. Experiment 2 includes two types of distractor arrangement: (a) a jumbled distractors' display as in Experiment 1 (that will require a serial attentive location check of the distractors) and (b) an organized distractors' display in which the distractors are arranged as a quadrilateral background. This quadrilateral arrangement is adapted from [32] where it has been shown that similar quadratic arrangement facilitated the grouping of distractors. Unlike the jumbled display, this arrangement facilitates the grouping of identical distractors since it follows several laws of grouping such as the law of proximity, the law of continuity, the law of good gestalt, and the law of symmetry and parallelism (see [33] for a review). Hence we predict that in the jumbled distractors' display, the same pattern of results as in Experiment 1 will be found. That is, RT for counting subset target items within the subitizing range will be faster with few distractors than with many distractors, for both distractors within the subitizing range and beyond the subitizing range. However, when distractors are organized in a specific pattern, allowing grouping, there will be no difference in RT for counting subset target items between few distractors and many distractors.

### Method

**Figure 4:**
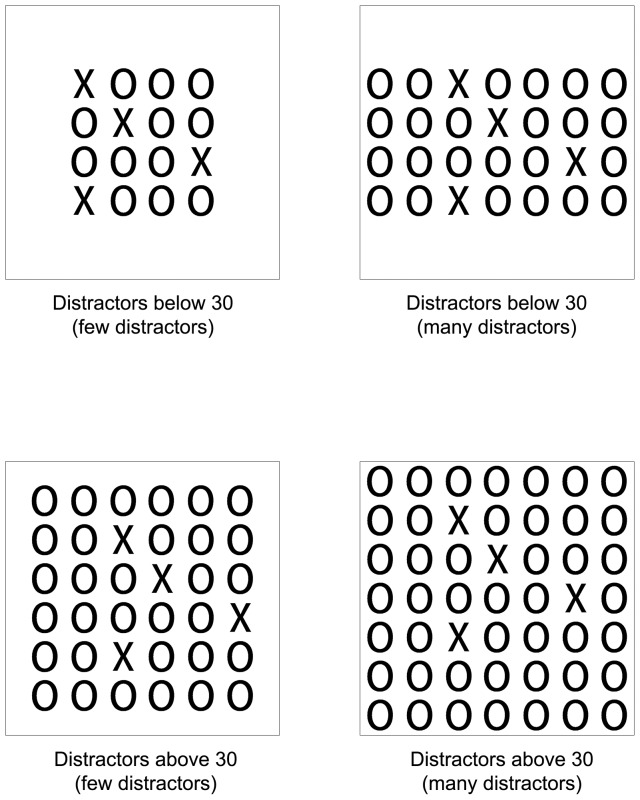
Examples of organized displays in the various conditions in Experiment 2.

The method in Experiment 2 was similar to that of Experiment 1 aside from the following changes. Sixteen participants took part in the experiment, with an age range of 20–34 (M = 26.12, SD = 3.54). Xs and Os sized approximately 0.8×0.5 were placed on an invisible 7×7 object grid sized with a similar size to the 6×6 grid of Experiment 1 (8.5×5 cm). As noted, this experiment included a jumbled display condition that was similar to the displays in Experiment 1. In addition this experiment also included an organized display condition that allowed distractor grouping. In this condition the distractors were arranged as a quadrilateral background using two types of distractors: distractors below 30 (small group) and distractors above 30 (large group). The distractors below 30 condition was similar in the amount of distractors to the beyond subitizing range condition in Experiment 1. In this condition each stimulus was constructed as an invisible grid filled with Os (distractors) and either 3 or 4 Xs (targets). The invisible grid was either 4×4 (few) or 4×7 (many) (see Figure 4). For each of those options (3 Xs in a 4×4 grid filled with Os, 3 Xs in a 4×7 grid filled with Os, 4 Xs in a 4×4 grid filled with Os, 4 Xs in a 4×7 grid filled with Os) 6 different versions were formed in which targets could appear in different locations on the grid. In total there were 24 different stimuli in this category. The distractors above 30 condition was similar to the distractors below 30 condition except for the number of distractors it contained. The purpose of displaying this condition was to verify that the results would not be restricted to a specific number range. In the distractors above 30 condition each stimulus was constructed as an invisible grid filled with Os (distractors) and either 3 or 4 Xs (targets). The invisible grid was either 6×6 (few) or 7×7 (many) (see Figure 4). For each of these options (3 Xs in a 6×6 grid filled with Os, 3 Xs in a 7×7 grid filled with Os, 4 Xs in a 6×6 grid filled with Os, 4 Xs in a 7×7 grid filled with Os) 6 different versions were formed in which targets could appear in different locations on the grid. In total there were 24 different stimuli in this category.

For both the distractors below 30 and the distractors above 30 conditions, for each one of the 6 versions the location of targets on the invisible grid remained the same for all types of distractors (4×4/4×7/6×6/7×7 grid). Overall, the experiment block contained 96 stimuli, each presented three times in randomized order (total of 288 stimuli).

### Results and Discussion

Trials in which participants didn't answer correctly (2.9%) and trials in which the RT was faster than 250 ms or slower than 2500 ms were not included in the analysis (0.2%).

For the remaining trials, the mean RT for each participant in each relevant condition was calculated. A three-way analysis of variance was applied to the mean RT with type of display (jumbled/organized), type of distractors (small group: subitizing range or distractors below 30/large group: beyond the subitizing range or distractors above 30), and number of distractors (few/many) as within participant factors.

There was a significant interaction between type of display and type of distractors *F*(1,15) = 4.82, *MS_e_* = 1451, *p<*.05 and between type of display and number of distractors *F*(1,15) = 15.13, *MS_e_* = 579, *p<*.01. A significant effect was also found for type of display (jumbled/organized), *F*(1,15) = 9.71, *MS_e_* = 2169, *p<*.01, and for type of distractors *F*(1,15) = 18.84, *MS_e_* = 4319, *p<*.01.

**Figure 5:**
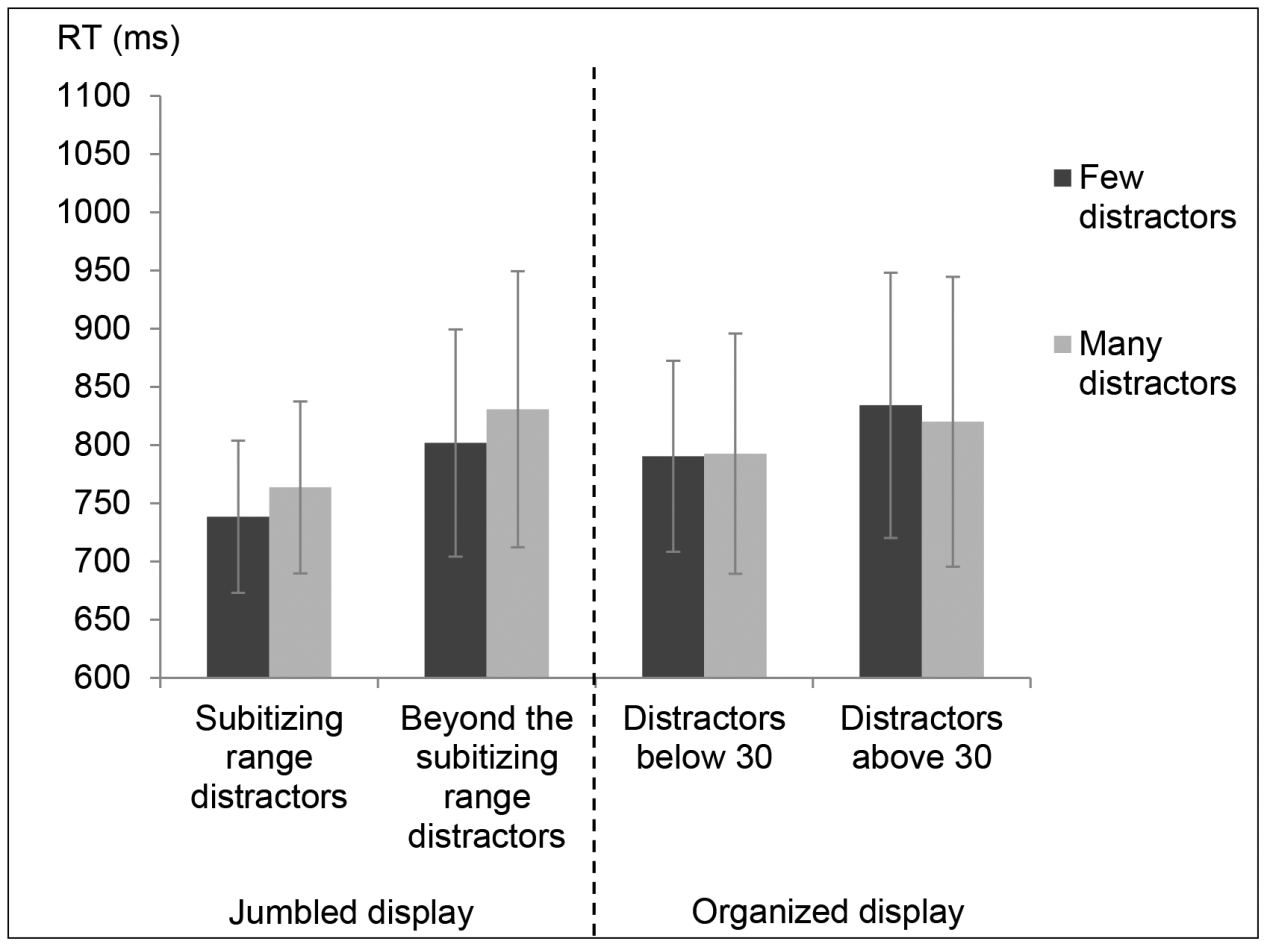
Mean RT and SD for counting subset items in the various conditions in Experiment 2.

Most importantly, further analysis revealed that as in Experiment 1 in the jumbled display, there was a significant effect for type of distractors (small group/large group), *F*(1,15) = 26.46, *MS_e_* = 2571, *p<*.001 and for number of distractors (few/many), *F*(1,15) = 17.85, *MS_e_* = 660, *p* = .001. There was no significant interaction (*F*<1). RT for counting subset target items within the subitizing range was significantly faster for few distractors (M = 738.29, SD = 65.4) than for many distractors (M = 763.68, SD = 73.88) [*t*(15) = −3.02, *p<*.01] in the small group. These results were also found in the large group condition in the jumbled display, as RT for few distractors (M = 801.75, SD = 97.5) was significantly faster than for many distractors (M = 830.67, SD = 118.65) [*t*(15) = −2.88, *p<*.05] (see figure 5). Overall, the results indicate that in the jumble display, RT for counting subset target items within the subitizing range became slower as the amount of distractors increased.

In contrast, in the organized display in the small group condition, RT for counting subset targets was not significantly faster for few distractors (M = 790.34, SD = 82.19) than for many distractors (M = 792.51, SD = 103.17) [*t*(15) = −.17, *n.s*]. These results were also found in the large group condition and RT for few distractors (M = 834.12, SD = 114.04) was in fact insignificantly slower than for many distractors (M = 820.02, SD = 124.62) [*t*(15) = 1.17, *n.s*] (see figure 5).

Overall, the results suggest that in a jumble display similar to Experiment 1, the amount of distractors interfered with the counting of a subset within the subitizing range. However, in an organized display, RT for counting subset target items within the subitizing range did not became slower as the amount of distractors increased.

Notably, one can claim that this pattern of results might derive from differences in crowding between the different conditions. One of the main characteristics used to define crowding is the density of the objects surrounding the targets [34]. One can assume that as the number of distractors increases, the display becomes more crowded and RT is slower. Hence it is possible that crowding may explain the finding that in the jumble displays RT was slower when the display contained more distractors. Although the different conditions in the jumbled display of Experiment 2 (which were the same conditions as in Experiment 1) might differ in their crowding, it is important to note that the crowding factor probably does not explain our current data. The reason for this is that it has been found that when targets and distractors differ from each other by a feature such as shape (as in the current study) the crowding of the distractors does not have a major effect on perception of the target [34]. However, in order to rule out the crowding effect we conducted the following analyses. In the first analysis we compared the RT in the display with 12 jumbled distractors (M = 801.75, SD = 97.5) (e.g., see Figure 2) to the 4×4 organized grid that had 12 or 13 distractors (M = 790.34, SD = 82.19) (e.g., see Figure 4). Note that the organized 4×4 display is more crowded than the jumbled display, such that if crowding was the explanation for our previous results then RT in the organized display should be slower than in the jumbled display. However the mean RT suggested an insignificant pattern in the opposite direction [*t*(15) = −.9, *n.s*]. Similarly we conducted another analysis in which we compared the display with 24 jumbled distractors (M = 816.21, SD = 106.72) (e.g., see Figure 2) to the 4×7 organized grid which had 24 or 25 distractors (M = 791.43, SD = 89.61), (e.g., see Figure 4). Once again, note that the organized 4×7 display is more crowded than the jumbled display, such that if crowding was the explanation for our previous results then RT in the organized display should be slower than in the jumbled display. Again, the mean RT did not support this assumption as it suggested a significant pattern in the opposite direction [*t*(15) = −3.09, *p<*.01]. Hence the crowding factor does not seem to explain our current result and in jumbled displays crowding cannot account for the increase in RT as the display contained more distractors.

## General Discussion

In conclusion, this study was designed to directly address the role of the number of distractors in subset enumeration. The study examined and compared different types of distractors: distractors within and outside the subitizing range, small and large amount of distractors, and examined the different arrangement of distractors in the display. In two experiments participants were asked to report the number of target items (Xs) among distractors (Os). Experiment 1 had two types of distractors: subitizing range distractors (few and many) and beyond the subitizing range distractors (few and many). The results demonstrated that RT for counting subset target items within the subitizing range was faster when there were few distractors compared to many distractors, both for distractors within the subitizing range and beyond it. Experiment 2 replicated this finding and also showed that when distractors were organized in a specific pattern, allowing grouping, an addition of distractors did not cause an increase in RT for counting subset target items.

The results indicate that when distractors are not grouped and subset counting is required, the number of subset items cannot be pulled out directly regardless of the number of other items in the display. The addition of distractors increases the cost in terms of RT.

It has been suggested [9] that it is possible to subitize items in an environment that contains distractors when the identities of the target and the distractors differ by a single feature (i.e., counting reds among greens or counting Xs among Os). However, subitizing is not possible when the identity of the targets differs by bound features (i.e., counting red Xs among red Os and green Xs). The current study suggests that even in order to perform a simple counting of a subset target (that differs from its distractors by a single feature), the enumeration process can begin only after the locations of the targets are identified and the targets' shape is bound to those locations. This pre-subitizing or pre-counting procedure depends on the number of individual locations occupied by the distractors. In a jumbled display, additional distractors added additional individual locations, resulting in increased RT. In contrast, in a grouped display such as the organized display condition in Experiment 2 - the additional distractors are perceived as part of the other distractors. The distractors are perceived as a single unit, group, or texture and additional distractors do not add additional individual locations. This, as predicted, does not result in RT increase as a simple function of the number of distractors as in this case there is no need for the attentional process to check each additional individual location.

In the introduction we mentioned several theories that may explain how subset numbers can be enumerated. The current results suggest that the number of a subset cannot be directly pulled out of the shape feature map without checking the location of each individual distractor. Similarly, the arithmetic system cannot directly count marked locations in an activation map without checking the location of each distractor. The results also suggest that the system cannot simply perceive the number of a subset as it perceives other features. This means that the number of a subset is not perceived quickly and independently similar to other features of the target such as the target's color or shape. Instead we suggest, based on the assumptions of the object file theory [22] (as described in Figure 1 - Theory 1), that perception of the number of subset items occurs only after the formation of a mental representation in which the individual locations and the shape of the items are already bound. According to this option, when identical subset items need to be enumerated the system must check each potential location in the environment and then match and bind the object's shape to its location. Only after this stage has been completed can the arithmetic system identify the number of target subsets. This option is compatible with the finding that even for targets within the subitizing range, the more individual distractors there are the more demanding is the perception of the targets' number.

## References

[1] Pepperberg IM (2006) Grey parrot (Psittacus erithacus) numerical abilities: Addition and further experiments on a zero-like concept. J Comp Psychol 120: 1–11. 16551159 10.1037/0735-7036.120.1.1

[2] Beran MJ (2007) Rhesus monkeys (Macaca mulatta) enumerate large and small sequentially presented sets of items using analog numerical representations. J Exp Psychol Anim Behav Process 33: 42–54. 17227194 10.1037/0097-7403.33.1.42

[3] Piazza M, Izard V, Pinel P, Le Bihan D, Dehaene S (2004) Tuning curves for approximate numerosity in the human intraparietal sulcus. Neuron 44: 547–555. 15504333 10.1016/j.neuron.2004.10.014

[4] Lipton JS, Spelke ES (2003) Origins of number sense: Large-number discrimination in human infants. Psychol Sci 14: 396–401. 12930467 10.1111/1467-9280.01453

[5] Kaufman EL, Lord MW, Reese TW, Volkmann J (1949) The discrimination of visual number. Am J of Psychol 62: 498–525. 15392567

[6] Butterworth B (1999). What counts: how every brain is hardwired for math. New York: Free Press. 274p.

[7] Mandler G, Shebo BJ (1982) Subitizing: An analysis of its component processes. J Exp Psychol Gen 111: 1–22. 6460833 10.1037//0096-3445.111.1.1

[8] Schleifer P, Landerl K (2011) Subitizing and counting in typical and atypical development. Dev Sci 14: 280–291. 22213901 10.1111/j.1467-7687.2010.00976.x

[9] Trick LM, Pylyshyn ZW (1993) What enumeration studies can show us about spatial attention: Evidence for limited capacity preattentive processing. J Exp Psychol Hum Percept Perform 19: 331–351. 8473843 10.1037//0096-1523.19.2.331

[10] Piazza M, Giacomini E, Le Bihan D, Dehaene S (2003) Single-trial classification of parallel pre-attentive and serial attentive processes using functional magnetic resonance imaging. Proc R Soc Lond B Biol Sci 270: 1237–1245. 10.1098/rspb.2003.2356 PMC1691365 12816636

[11] Egeth HE, Leonard CJ, Palomares M (2008) The role of attention in subitizing: Is the magical number 1? Vis Cogn 16: 463–473.

[12] Poiese P, Spalek TM, Di Lollo V (2008) Attentional involvement in subitizing: Questioning the preattentive hypothesis. Vis Cogn 16: 474–485.

[13] Vetter P, Butterworth B, Bahrami B (2010) A Candidate for the attentional bottleneck: Set-size specific modulation of right TPJ during attentive enumeration. J Cogn Neurosci 23: 728–736. 20350059 10.1162/jocn.2010.21472

[14] Olivers CNL, Watson DG (2008) Subitizing requires attention. Vis Cogn 16: 439–462.

[15] Burr DC, Turi M, Anobile G (2010) Subitizing but not estimation of numerosity requires attentional resources. J Vis 10: 1–10. 10.1167/10.6.20 20884569

[16] Goldfarb L, Treisman A (2013) Subset enumeration and the lack of binding errors. Manuscript submitted for publication.

[17] Treisman A, Gelade G (1980) A feature integration theory of attention. Cogn Psychol 12: 97–136. 7351125 10.1016/0010-0285(80)90005-5

[18] Treisman A, Schmidt H (1982) Illusory conjunctions in the perception of objects. Cogn Psychol 14: 107–141. 7053925 10.1016/0010-0285(82)90006-8

[19] Treisman A (2006) How the deployment of attention determines what we see. Vis Cogn 14: 411–443. 17387378 10.1080/13506280500195250 PMC1832144

[20] Halberda J, Mazzocco M, Feigenson L (2008) Individual differences in non-verbal number acuity correlate with maths achievement. Nature 455: 665–668. 18776888 10.1038/nature07246

[21] Goldfarb L, Treisman A (2013) Counting multidimensional objects: Implications for the neural-synchrony theory. Psychol Sci 24: 266–271. 23334446 10.1177/0956797612459761

[22] Kahneman D, Treisman A, Gibbs BJ (1992) The reviewing of object files: Object-specific integration of information. Cogn Psychol 24: 175–219. 1582172 10.1016/0010-0285(92)90007-o

[23] Huang L, Treisman A, Pashler H (2007) Characterizing the limits of human visual awareness. Science 317: 823–825. 17690299 10.1126/science.1143515

[24] Wolfe JM (1994) Guided Search 2.0: A revised model of visual search. Psychon Bull Rev 1: 202–238. 24203471 10.3758/BF03200774

[25] Feldman J (1999) The role of objects in perceptual grouping. Acta Psychol 102: 137–163. 10.1016/s0001-6918(98)00054-7 10504879

[26] Scholl BJ (2001) Object and attention: the state of the art. Cognition 80: 1–46. 11245838 10.1016/s0010-0277(00)00152-9

[27] Rock L, Palmer S (1990) The legacy of Gestalt psychology. Sci Am 263: 48–61. 10.1038/scientificamerican1290-84 2270461

[28] Ward R, Goodrich S, Driver J (1994) Grouping reduces visual extinction: Neuropsychological evidence for weight-linkage in visual selection. Vis Cogn 1: 101–129.

[29] Gilchrist I, Humphreys GW, Riddoch MJ (1996) Grouping and extinction: Evidence for low-level modulation of selection. Cogn Neuropsychol 13: 1223–1256.

[30] Goldfarb L, Treisman A (2011a) Repetition blindness: The survival of the grouped. Psychon Bull Rev 18: 1042–1049. 21811897 10.3758/s13423-011-0135-4

[31] Goldfarb L, Treisman A (2011b) Does a color difference between parts impair the perception of a whole? A similarity between Simultanagnosia patients and healthy observers. Psychon Bull Rev 18: 877–882. 21748421 10.3758/s13423-011-0123-8

[32] Treisman A (1982) Perceptual grouping and attention in visual search for features and for objects. J Exp Psychol Hum Percept Perform 8: 194–214. 6461717 10.1037//0096-1523.8.2.194

[33] Wagemans J, Elder JH, Kubovy M, Palmer SE, Peterson MA, et al (2012) A century of Gestalt psychology in visual perception: I. Perceptual grouping and figure–ground organization. Psychol Bull 138: 1172–1217. 22845751 10.1037/a0029333 PMC3482144

[34] Whitney D, Levi DM (2011) Visual crowding: A fundamental limit on conscious perception and object recognition. Trends Cogn Sci 15: 160–168. 21420894 10.1016/j.tics.2011.02.005 PMC3070834

